# Reactive surface organometallic complexes observed using dynamic nuclear polarization surface enhanced NMR spectroscopy†
†Electronic supplementary information (ESI) available: Preparation of the samples; nitrogen absorption/desorption isotherms, FT-IR spectroscopy, DNP SENS spectroscopy and DFT calculations. See DOI: 10.1039/c6sc02379g



**DOI:** 10.1039/c6sc02379g

**Published:** 2016-08-15

**Authors:** Eva Pump, Jasmine Viger-Gravel, Edy Abou-Hamad, Manoja K. Samantaray, Bilel Hamzaoui, Andrei Gurinov, Dalaver H. Anjum, David Gajan, Anne Lesage, Anissa Bendjeriou-Sedjerari, Lyndon Emsley, Jean-Marie Basset

**Affiliations:** a King Abdullah University of Science and Technology (KAUST) , KAUST Catalysis Center (KCC) , Thuwal , 23955-6900 , Saudi Arabia . Email: jeanmarie.basset@kaust.edu.sa ; Email: anissa.bendjeriousedjerari@kaust.edu.sa; b Institut des Sciences et Ingénierie Chimiques , Ecole Polytechnique Fédérale de Lausanne (EPFL) , CH-1015 Lausanne , Switzerland . Email: lyndon.emsley@epfl.ch; c Imaging and Characterization Lab. King Abdullah University of Science and Technology (KAUST) , Thuwal , 23955-6900 , Saudi Arabia; d Institut de Sciences Analytiques (CNRS/ENS-Lyon/UCB-Lyon 1) , Université de Lyon , Centre de RMN à Très Hauts Champs , 69100 Villeurbanne , France

## Abstract

Dynamic Nuclear Polarization Surface Enhanced NMR Spectroscopy (DNP SENS) is an emerging technique that allows access to high-sensitivity NMR spectra from surfaces. However, DNP SENS usually requires the use of radicals as an exogenous source of polarization, which has so far limited applications for organometallic surface species to those that do not react with the radicals. Here we show that reactive surface species can be studied if they are immobilized inside porous materials with suitably small windows, and if bulky nitroxide bi-radicals (here TEKPol) are used as the polarization source and which cannot enter the pores. The method is demonstrated by obtaining significant DNP enhancements from highly reactive complelxes [(≡Si–O–)W(Me)_5_] supported on MCM-41, and effects of pore size (6.0, 3.0 and 2.5 nm) on the performance are discussed.

## Abstract 2

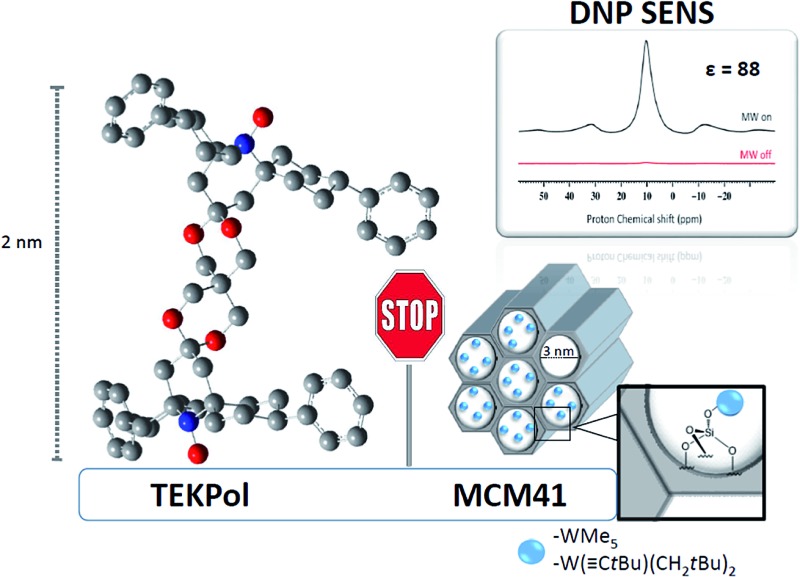
Reactive surface species immobilized inside porous materials with suitably small windows can be studied by DNP SENS.

## Introduction

Heterogeneous catalysis is ubiquitous today and is central to solving many of the key problems facing chemistry including energy and environmental issues that contribute to a sustainable world.^1,2^ However, many heterogeneous catalysts contain numerous types of active sites which makes it difficult to reflect the intrinsic efficiency of the catalyst. To develop structure–activity relations that would lead to “Catalysis by Design”, Surface Organometallic Chemistry (SOMC) has been introduced to provide a single-site strategy^3–5^ by creating well defined surface organometallic fragments (SOMF) that are presumed to be part of the catalytic cycle.^5^ To achieve this goal, the surface complexes need to be unambiguously characterized, usually through advanced spectroscopic techniques such as FT-IR, EXAFS and multi-dimensional solid-state NMR spectroscopy. By combining these tools, structure–activity relationships can be determined.^4,5^ For example, in the discovery of alkane metathesis^6^ over tantalum hydride, and its subsequent improvement^7,8^ with [(≡Si–O–)W(Me)_5_] supported on silica, solid-state NMR spectroscopy in particular proved to be an essential technique to characterise surface structures obtained using the SOMC methodology.^9–12^ However, the low sensitivity of NMR is a major handicap. As an illustration, the identification of surface carbene or carbyne (Ta or W) complexes using ^13^C CP MAS NMR spectroscopy is challenging due to a low signal-to-noise ratio, and this despite acquisition times for one-dimensional spectra that can be several days.^7,8,13,14^ To address these issues, the most common route is to introduce carbon-13 enriched complexes, ideally selectively labelled at the α position of the metal center, but the synthesis of such compounds is extremely difficult and time consuming. Dynamic nuclear polarization surface enhanced NMR spectroscopy (DNP SENS) has been recently introduced to solve these sensitivity concerns.^17–22^ In these experiments the material is impregnated with a solution containing stable free radicals (usually bi-nitroxides) and polarization is transferred from the unpaired electrons of the radical to the surrounding nuclei (usually protons) by *in situ* microwave (μwave) irradiation followed by spin diffusion and cross-polarisation (CP) to the nuclei of interest.^22^ On commercial instruments, this is performed at temperatures around 100 K and under magic angle spinning (MAS) conditions (typically at 8–12 kHz). This recently introduced method has been very successful for the characterization of a broad panoply of materials ranging from inorganic materials to pharmaceuticals and organic materials.^15–30^

However, so far the method is problematic when being applied to surface complexes on silica that react with the radicals. This precludes the characterization of some of the most interesting catalytic species. There is thus a need for a non-destructive strategy for the DNP SENS characterization of SOMC complexes. An important exception has been observed where reactive zirconium amides immobilized on mesoporous silica did not react with the nitroxyl radicals in DNP experiments, although they would normally be expected to do so.^31^

Here, we propose a new strategy which is based on avoiding direct contact between the active catalytic site and the nitroxide radical by: (a) immobilizing the surface complexes inside mesoporous materials with small windows and, (b) using bulky radicals which presumably will not enter the cavities, but could transfer their polarization through the solvent which could be small enough to penetrate inside the mesopores. Indeed, polarization relayed by spin diffusion has already been observed in porous systems when the radicals were excluded by size effects.^29,30,32^

As a proof of concept, we investigate here two types of highly sensitive supported tungsten complexes: W(≡C*t*Bu)(CH_2_ *t*Bu)_3_ (**A**)^14^ and W(Me)_6_ (**B**)^7^ on different mesoporous materials (SBA-15 and MCM-41) using DNP SENS. Fine-tuning of the mesoporous pore diameter (6.0, 3.0 and 2.5 nm) allows us to preserve the integrity of the catalytic sites by sterically excluding the large biradical nitroxide TEKPol.^33^ The surface complexes are polarized by spin diffusion from the radical through the impregnating solution in the pores, and we obtain signal enhancement factors (*ε*, which is defined as the ratio of signal intensities of spectra acquired with and without microwave irradiation) of up to 30, corresponding to a reduction in experimental acquisition time by a factor of 900.

## Results and discussion

**Fig. 1:**
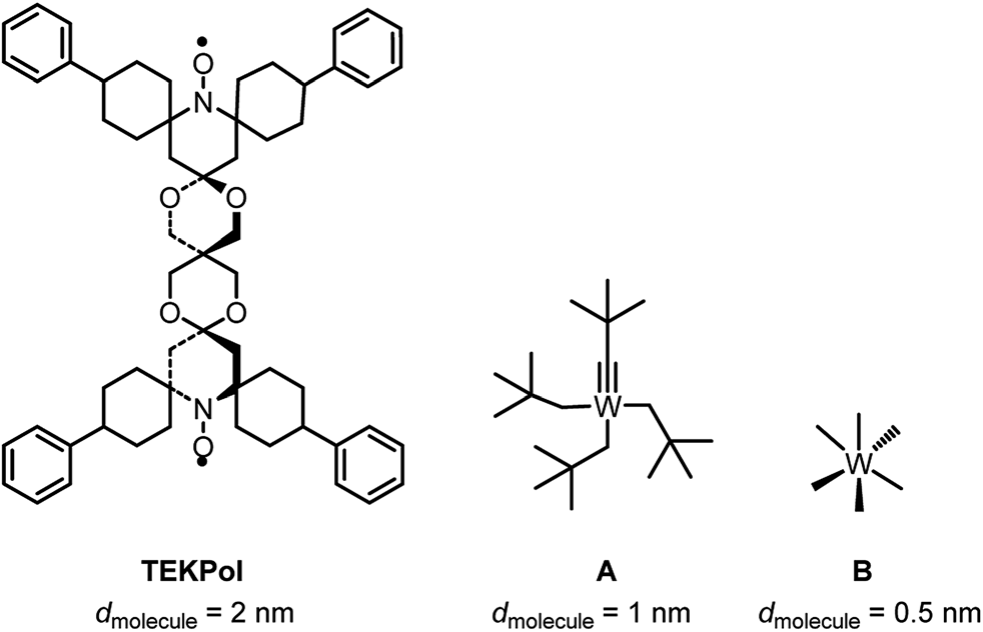
Chemical structure and size (*d*_molecule_) of TEKPol, W(≡C*t*Bu)(CH_2_ *t*Bu)_3_ (**A**) and W(Me)_6_ (**B**) based on DFT calculations (ESI†).

Given the size of TEKPOL (*d*_molecule_ = 2 nm) and of the organometallic complexes W(≡C*t*Bu)(CH_2_ *t*Bu)_3_ **A** (*d*_molecule_ = 1 nm) and WMe_6_ **B** (*d*_molecule_ = 0.5 nm) (Fig. 1), mesoporous materials with different pore sizes (*d*_pore_) 6.0 nm (SBA-15),^34^ 3.0 and 2.5 nm (MCM-41)^35,36^ were prepared (ESI†). The objective was to develop materials that have large enough pores to accommodate the surface species, but small enough to prevent entry of the radical.

The dehydroxylation process of the support was performed at 500 °C under high vacuum (10^–5^ mbar) for 16 hours and led to the formation of isolated silanols (≡SiOH).^37^ Under these experimental conditions, MCM-41 is prevented from collapsing^38^ and the well-ordered hexagonal structure of each material remains intact as shown by N_2_ sorption, small angle X-ray diffraction (XRD) and transmission electron microscopy (TEM) (Fig. S2 and S3, Table S2, ESI†).

**Scheme 1:**
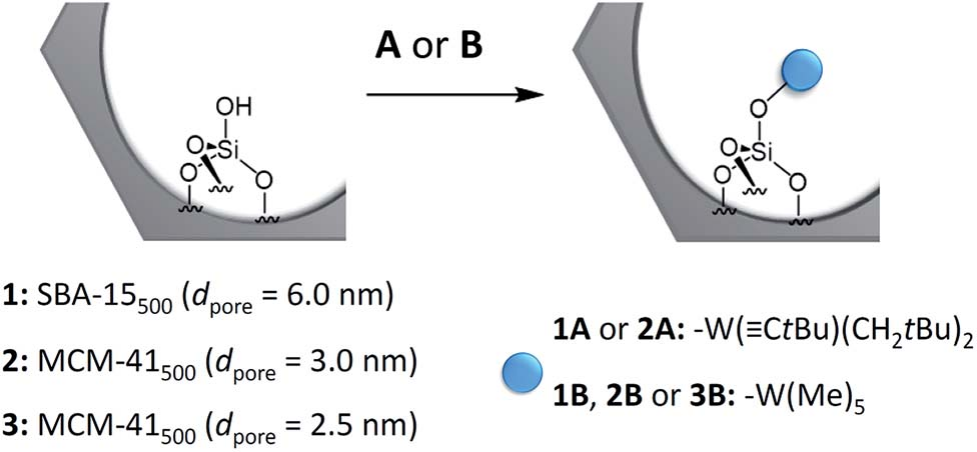
Generalized scheme for the reaction of single silanols on mesoporous materials with complex W(≡C*t*Bu)(CH_2_ *t*Bu)_3_ (**A**), or W(Me)_6_ (**B**).

Scheme 1 shows the schematic reaction of **A** and **B** with the ≡SiOH groups of SBA-15_500_ (**1**) and MCM-41_500_ (**2** and **3**) as previously described in the literature.^8,14^ Elemental analysis revealed the formation of the monopodal surface complexes [(≡SiO–)W(≡C*t*Bu)(CH_2_ *t*Bu)_2_] (**1A** and **2A**) and [(≡SiO–)W(Me)_5_] (**1B**, **2B** and **3B**) (Table S3, ESI†). The FT-IR, ^1^H and ^13^C CP MAS NMR spectra obtained are in accordance with previous work.^5,8,14^

DNP SENS measurements of all the surface organometallic complexes were performed using incipient wetness impregnation^39^ in a glove box. In a typical experiment, a 15 mg sample was impregnated with the appropriate volume of 16 mM TEKPol in 1,1,2,2-tetrachloroethane (TCE) or 1,2-dichlorobenzene (DCB) (ESI†). As reported in the literature, the best enhancement for applications requiring organic solvents were obtained with TEKPol^33^ in combination with organic solvents such as TCE and DCB.^40^ DCB was preferred here, since its carbon resonances (*δ* = 127–132 ppm) do not overlap with the expected ^13^C chemical shifts of both the SOMC catalysts studied here. DNP experiments were performed at 100 K with MAS spinning rates varying between 8–12 kHz. All observed enhancements are summarized in Table 1. As a control, we immobilized W(≡C*t*Bu)(CH_2_ *t*Bu)_3_ (**A**) on nonporous silica SiO_2–700_ particles (BET surface area = 183 m^2^ g^–1^).^41^ The resulting surface organometallic complex **0A** displays a typical solid state ^13^C CP MAS NMR spectrum of a monopodal single supported non-^13^C enriched tungsten [(≡SiO–)W(≡C*t*Bu)(CH_2_ *t*Bu)_2_].^14^ This spectrum was acquired without TEKPol solution impregnation and without DNP and it required a number of scans of 60 000 (Fig. S1, ESI†). Then, we tried to characterize **0A** using DNP SENS. However, poor proton and carbon enhancements [*ε*_H_(DCB) is 4.8(0.2)] and no surface signals were obtained, confirming, as expected, the reactivity of the W-fragment towards TEKPol .

**Table 1:** ^1^H and ^13^C enhancements of the surface complexes **0A**, **1A**, **1B**, **2A**, **2B** and **3B** impregnated with a 16 mM TEKPol solution in either DCB or TCE. The spectra were recorded with a recycle delay of 3 s after sample preparation if not noted differently

Sample	Solvent	*ε*_H_ (solvent)	*ε*_C,CP_ (surface)
**0A**	DCB	4.8(0.2)	—
**1A** ^*a*^	DCB	1.98(0.01)	—
**1B**	DCB	12.96(0.03)	—
**2A**	DCB	11.2(1.1)	—
**2A**	TCE	28.7(0.7)	—
**2B**	DCB	59.1(0.1)	31.2(0.1)
**3B**	DCB	85.2(0.5)	5.5(0.3)
**3B** ^*b*^	DCB	20.4(0.2)	15.2(0.4)

^*a*^The sample was left to impregnate at –4 °C for 4 hours.

^*b*^The sample was left to impregnate at –4 °C for 21 days.

Similarly, catalysts grafted on SBA-15_500_ (*d*_pore_ = 6.0 nm) also showed almost no solvent enhancement in both cases, **1A** and **1B.** Solvent enhancements were around 5–10, and further decreased after 4 h (to around 2). SBA-15 has been used on several occasions for DNP SENS of non-reactive materials, and enhancements above 100 (in DCB) and 250 (in TCE) were reported. Our results indicate that TEKPol diffuses, at least partially, inside the mesopores where it probably rapidly reacts with the single site catalyst to form an organometallic nitroxide complex which is thus inactive for DNP.^42–44^

To prevent the reaction of the surface catalyst with the radical we used MCM-41 as a support with a pore size *d*_pore_ = 3.0 nm (**2**).^36^ Despite the smaller pore size, both **A** and **B** can be successfully grafted onto **2**.

**Fig. 2:**
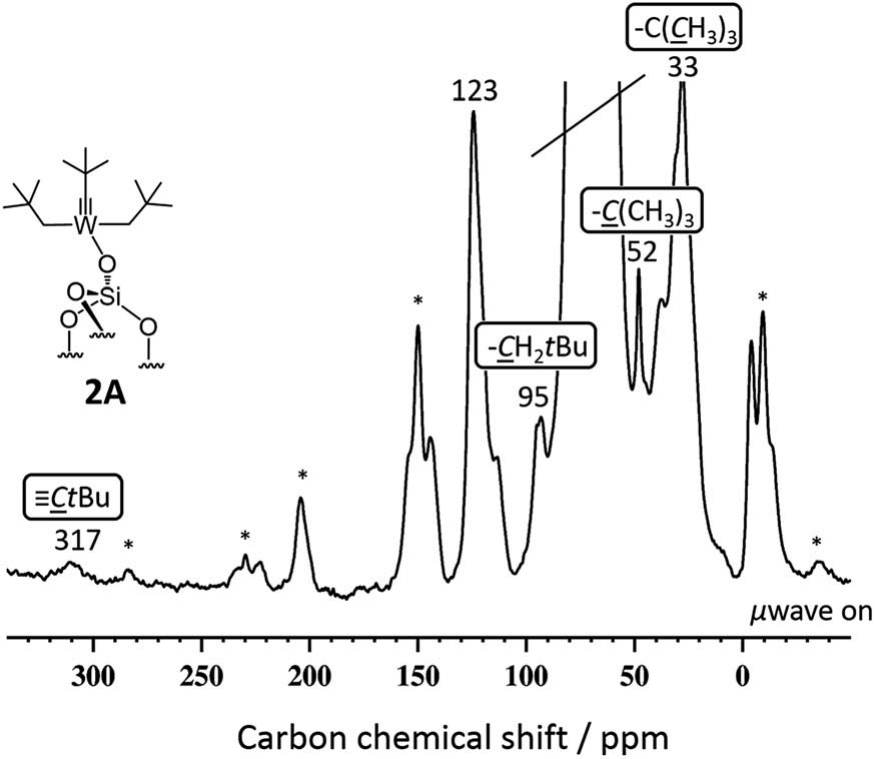
^13^C CP MAS DNP SENS spectra (100 K, 400 MHz/263 GHz) of **2A** in a 16 mM TEKPol solution in TCE. The recycle delay was 3 s, the contact time was 3 ms and the MAS frequency was 8 kHz. All characteristic resonances were obtained after 8000 scans. The signal at 123 ppm corresponds to reacted TEKPol, the stars indicate the spinning side bands.

[(≡SiO)–W(≡C*t*Bu)(CH_2_ *t*Bu)_2_] **2A** gave enhancements in proton and carbon experiments using either DCB or TCE [*ε*_H_(DCB) = 11.2(1.1), *ε*_H_(TCE) = 28.7(0.7)]. More importantly, the DNP SENS ^13^C CP MAS of **2A** showed all the expected characteristic peaks of the surface complexes at 32, 52, 95 and 317 ppm corresponding to the [W–CH_2_C(CH_3_)_3_], [W–CH_2_C(CH_3_)_3_], [W–CH_2_C(CH_3_)_3_] and [W≡C–C(CH_3_)_3_], respectively (Fig. 2). This spectrum was recorded with 8000 scans with a S/N ratio of 20(2) at 95 ppm (see Fig. 2). For comparison, in conventional NMR spectroscopy only the signals at 32, 52 and 95 ppm (the carbyne was not observed) were found after 70 000 scans with a poor signal-to-noise ratio of 4(1) of the same signal.^14^ Around 10% of the grafting occurs at the readily accessible outer surface of the particle (Table S2, ESI†). We believe that the lower than usual enhancement is due to the partial reaction of TEKPol with the active sites of **2A** at the external surface and at the pore entry.

The second material **2B**, with a diameter of the molecular fragment of 0.5 nm, led to better results in comparison to **2A**. DNP SENS ^1^H MAS, ^13^C and ^29^Si CP MAS spectra were acquired with good quality [Table 1, *ε*_H_(DCB) = 59.1(0.1), *ε*_C,CP_(**2B**) = 31.2(0.1) and *ε*_Si,CP_(**2B**) = 76.8(0.7)] in a few scans (16, 512 and 1024 scans, respectively). More detailed information can be obtained in Table S4, ESI.†

**Fig. 3:**
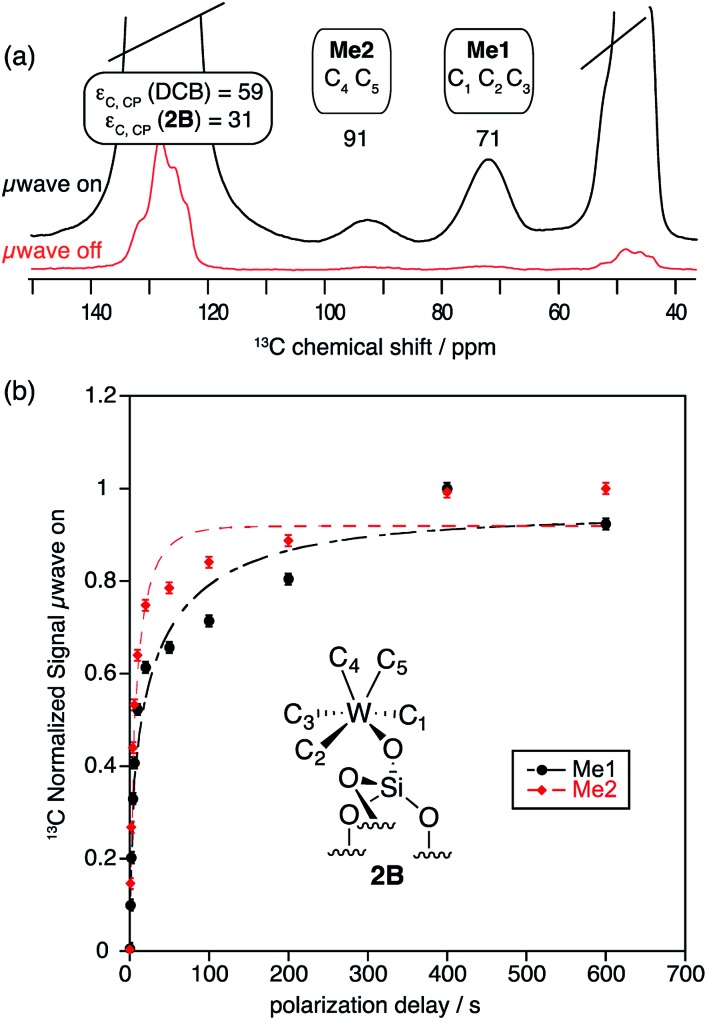
^13^C CP MAS DNP SENS (100 K, 400 MHz, 263 GHz gyrotron) of 2B in 16 mM TEKPol DCB solution. The acquisition parameters are: 3 s recycle delay, 3 ms contact time and it was acquired with 512 scans at 8 kHz MAS frequency. In (a) are the ^13^C CP MAS spectra acquired with microwave on (black trace) and off (red trace); and (b) the ^13^C normalized signal build up as a function of polarization times for both types of –CH_3_ groups with microwave irradiation. Data were fit using a stretched-exponential function. The stretched-exponential function has the following form: 
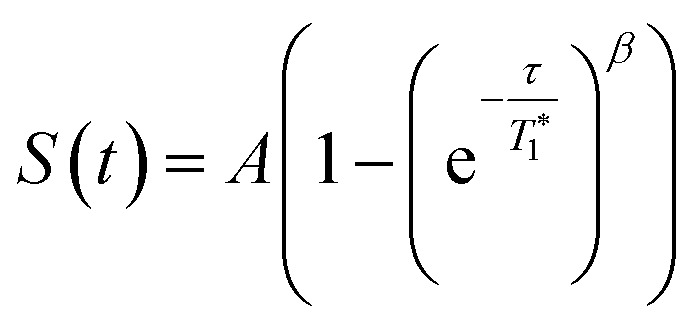
, where *A* is the equilibrium normalized signal, *β* is the stretched parameter, *τ* is the polarization time and 
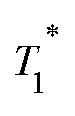
 is the observed signal build-up time. The stretched exponential character is a signature of relayed DNP transfer.^46^

As expected from previous low temperature SS NMR studies of [(≡Si–O)–(WMe_5_)],^8^ the ^13^C CP MAS DNP SENS spectrum of **2B** acquired at 100 K shows the two non-equivalent types of methyl groups [C_1_, C_2_, C_3_ (**Me1**) and C_4_, C_5_ (**Me2**)] at 71 and 91 ppm, respectively (Fig. 3a and Fig. S8, ESI†), after only 512 scans which corresponds to a time saving of a factor of 970. Note that a factor of 3 in the sensitivity gain is due to the fact that DNP experiments are conducted at low temperature.^45^ In comparison, the conventional ^13^C CP MAS spectrum recorded at RT shows only one signal at 82 ppm after 43 000 scans, due to coalescence at room temperature.^8^

To obtain more information about the mechanism of polarization of complexes inside MCM-41 **2** in the DNP SENS experiments, we conducted a ^13^C saturation recovery experiment for both types of methyl groups (**Me1** and **Me2**) of **2B**, shown in Fig. 3. The measurements were then fit with a stretched-exponential function as shown in Fig. 3 (Table S5, ESI†). The data fit reasonably well and in particular the pronounced multi-exponential character of the polarization build-up is a signature of DNP relayed by spin diffusion, as has been seen previously in micro-crystalline solids, metal–organic frameworks and zeolites.^27,30,32,46^

The greatly increased sensitivity obtained here allows for experiments within short acquisition times that were previously hardly conceivable on these types of reactive surface to provide much more precise and detailed characterization of the complexes. For example, to obtain a ^29^Si CP MAS NMR spectrum with the same quality as obtained by this DNP SENS experiment, the time saving translates to a factor of around 6000. The ^29^Si CP MAS DNP SENS spectrum of **2B** was acquired with only 1024 scans (compared to 20 000 scans in conventional NMR experiments)^8^ with an enhancement of *ε*_Si,CP_ = 76.8(0.7) (Fig. S9, ESI†). The spectrum consists of one major peak at –100 ppm indicating that most silicon atoms are present as Q^4^ and Q^3^-sites,^20,48^ and most interestingly a weak signal at –16 ppm is assigned to methyl-transfer to Si, arising from the formation of bipodal species, which can occur at room temperature.^49^

We were also able to perform DNP SENS 2D experiments. As an example, a two-dimensional (2D) ^1^H–^13^C dipolar HETCOR NMR spectrum of **2B** was recorded (Fig. S10, ESI†). As expected, the 2D spectrum shows two correlations with different ^1^H chemical shifts for each methyl group (**Me1**: *δ*(^1^H) = 2.1 ppm and *δ*(^13^C) = 92 ppm, and **Me2**: *δ*(^1^H) = 1.7 ppm and *δ*(^13^C) = 72 ppm).^8^

**Fig. 4:**
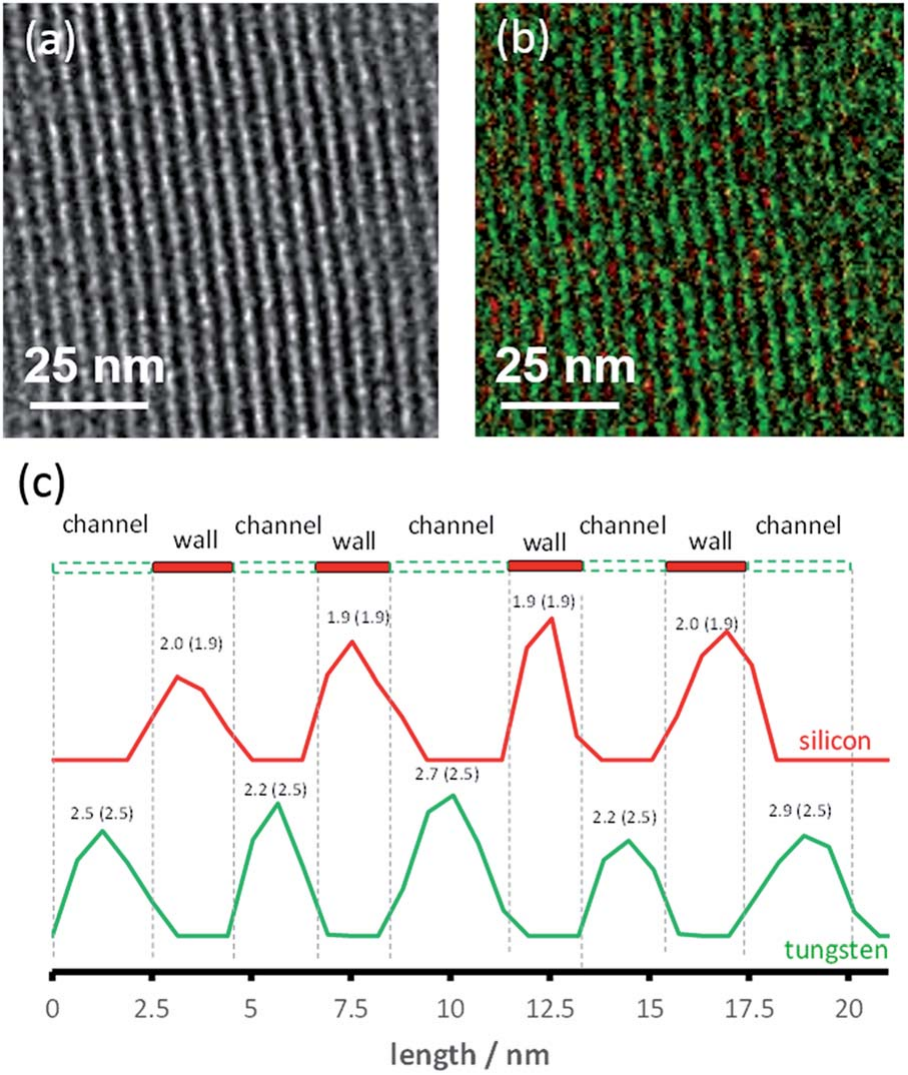
(a) BF-TEM analysis of **3B** showing the structure of the sample; (b) superimposed Si (red) and W (green) elemental maps of **3B** acquired by applying EF-TEM to the Si L_2–3_ and W_4–5_ edges. The W content seems to be higher in comparison to Si because the O_4–5_ edge of W is close to the zero-loss peak; (c) line profile confirms the wall and channel width dimensions. The counts of silicon and tungsten were normalized to 1. Observed values for channels and walls are compared to BET and small angle XRD values in parentheses.

A second MCM-41 with a smaller pore size (2.5 nm) (**3**) was also synthesized and used as a support. Both organometallic complexes **A** and **B** were grafted on material **3**. However, only **3B** gave reliable results in the DNP experiments, likely due to its smaller molecular diameter; the SOMF of **3A** is likely only present on the “external” surface. The successful grafting of WMe_6_ **B** inside the mesopores of **3** is confirmed by BJH sorption results and a reduction in the pore size by 0.7 Å. TEM showed that the hexagonal structure of the material was maintained (Fig. 4a). Energy filtered (EF)-TEM at the energy losses of 35 eV (O_4–5_-edge of W) and 99 eV (L_2–3_-edge of Si) were used to obtain respective elemental maps of W and Si. Bright-field TEM (BF-TEM) filtered images of several particles were acquired by inserting the energy slit of 20 eV width around the zero-loss peak and a representative image is shown in Fig. 4a. A superimposed map generated from W and Si is shown in Fig. 4b and it confirms that the metal complex (W = green) is well distributed inside the channels of the mesoporous material (Si = red) (Fig. 4b). Moreover, the line profile analysis (Fig. 4c) allowed the determination of the widths of the walls (1.9 nm) and channels (2.5 nm) of the mesoporous material. These results are in agreement with the nitrogen sorption and small angle XRD results.

One dimensional DNP SENS ^1^H MAS and ^13^C CP MAS spectra of **3B** yielded high solvent enhancements for DCB (*ε*_H_ = 85.2(0.5)) while a much lower enhancement was observed [*ε*_C,CP_(**3B**) = 5.5(0.3)] for the characteristic surface Me-signals of **3B** at 71 and 91 ppm. This lower enhancement is again a signature of the polarization process being relayed by spin diffusion inside the pores.^27,30,46^ It is particularly interesting to note that we observe changes in the DNP behaviour with time (Table 1 and Fig. S11, ESI†). This most likely indicates that the pores are not fully impregnated immediately, and that diffusion of the solvent into the materials can continue over a period of days^49^ (a similar behavior was also observed for MOFs^27^).

**Fig. 5:**
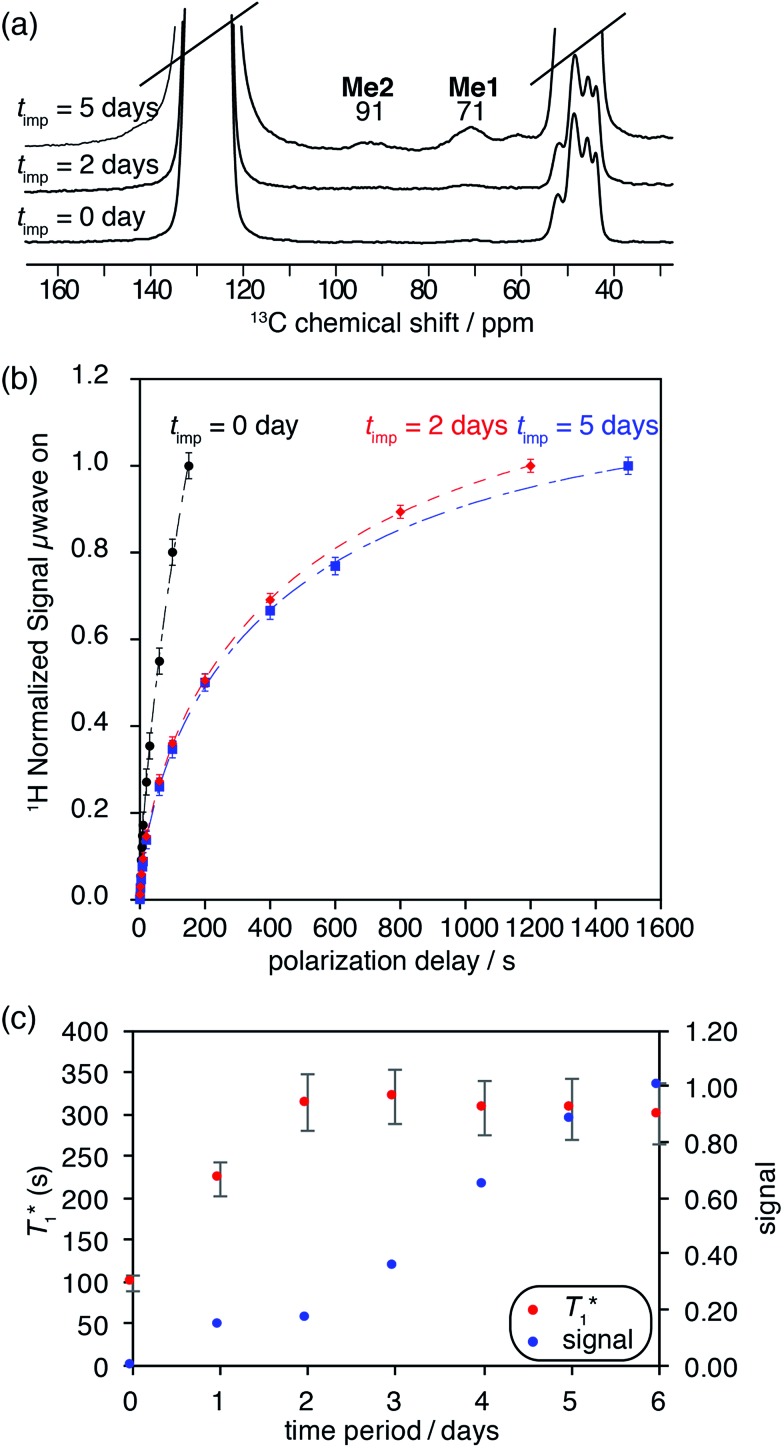
(a) ^13^C CP MAS DNP SENS spectra (100 K, 400 MHz/263 GHz) of **3B** in 16 mM TEKPol DCB solution, acquired with the following parameters: a recycle delay of 3 s, a contact time of 3 ms and 8 kHz MAS frequency. The spectra were acquired with microwave on for compound **3B** at different time intervals (indicated on the spectra). Between each experiment the sample was stored in its rotor at –4 °C. In (b) are the ^1^H normalized signal build-up curves as a function of time acquired at various impregnation times, with microwave irradiation. (c) Measured 
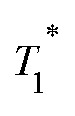
 (^1^H) values as well as the signal intensity of the surface (**Me2**) (measured for a recycle delay of 3 s) for compound **3B** as a function of the impregnation time (monitored over one week and sampled every day) are shown by red and blue circles, respectively.

A series of ^1^H saturation recovery curves were measured for this sample over a period of one week. Each build-up curve was then fit with a mono-exponential function (Table S6, ESI†). DNP SENS spectra and the saturation curves at impregnation time (*t*_imp_) = 0 days (immediately after sample preparation), 2 days and 5 days are illustrated in Fig. 5a and b. The ensemble of DNP parameters (
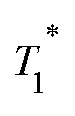
 and signal intensity) is shown as a function of the impregnation time in Fig. 5c. The ^1^H and ^13^C enhancement for compound **3B** for each time period can be found in Table S6 in the ESI.†

At *t*_imp_ = 0, fast solvent build-up times and a concomitant high solvent enhancement was observed (ESI†). Only weak characteristic resonances of SOMC **3B** were obtained, because the main part of the solvent volume is outside the mesopores or at the pore entry. With time, the 
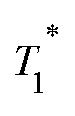
 of the solvent increases over a period of three days and becomes constant. Also, the signal intensities of the methyl groups of the surface species in compound **3B** are observed to increase over the course of one week. These changes suggest that the pores are slowly filled up with DCB. Thus, a character consistent with relayed polarization is observed. After 5 days, DCB is distributed throughout the mesopores. The polarization can be transferred by spin diffusion through the solvent to **3B** leading to increased signal from the surface complex **3B**.

## Conclusions

In conclusion, we have demonstrated that using MCM-41 with small pore sizes (2.5–3.0 nm) as a solid support for reactive organometallic surface species enables characterization using DNP SENS. This strategy is based on the design of a support with an adequate pore size in order to (a) avoid direct contact between TEKPol and the active site, (b) maintain good spin diffusion within a reasonable time, and (c) be large enough to accommodate the small to medium sized surface species studied here. The large DNP enhancements obtained enable rapid characterization including the acquisition of multi-dimensional spectra that would have been unfeasible without the approach introduced here.
