# Supplementary material for: Reactive surface organometallic complexes observed using dynamic nuclear polarization surface enhanced NMR spectroscopy
Source: Chem Sci. 2016 Aug 15;8(1):284–90. doi: 10.1039/c6sc02379g (PMC5365068; doi:10.1039/c6sc02379g)
Supplement: Supplementary file 1 [file SC-008-C6SC02379G-s001.pdf]

Electronic Supplementary Information

Reactive Surface Organometallic Complexes Observed by Dynamic Nuclear Polarization Surface Enhanced NMR Spectroscopy

Eva Pump,[a] Jasmine Viger-Gravel,[b] Edy Abou-Hamad,[a] Manoja K. Samantaray,[a] Bilel Hamzaoui,[a] Andrei Gurinov,[c] Dalaver H. Anjum,[c] David Gajan,[d] Anne Lesage,[d] Anissa Bendjeriou-Sedjerari,*[a] Lyndon Emsley*[b] and Jean-Marie Basset*[a]

[a] KAUST Catalysis Center (KCC), King Abdullah University of Science and Technology, Thuwal-23955-6900, Saudi Arabia,

[b] Institut des Sciences et Ingénierie Chimiques, Ecole Polytechnique Fédérale de Lausanne (EPFL), CH-1015 Lausanne, Switzerland,

[c] Imaging and Characterization Lab. King Abdullah University of Science and Technology (KAUST), Thuwal, 23955-6900, Saudi Arabia

[d] Institut de Sciences Analytiques (CNRS / ENS-Lyon / UCB-Lyon 1), Université de Lyon, Centre de RMN à Très Hauts Champs, 69100 Villeurbanne, France

Table of contents

1. General Procedure 2

2. Nitrogen adsorption/desorption isotherms 8

3. IR spectra 10

4. DNP SENS 11

5. DFT calculations 16

1. General Procedure

All experiments were carried out under controlled atmosphere. Treatments of the surface species were carried out using high vacuum lines (1.34 Pa) and glove box techniques. *n*-Pentane was distilled on Na - benzophenone and degassed through freeze pump thaw cycles.

__Typical procedure of synthesis of mesoporous material__

Synthesis of SBA-15 with 6.0 nm pore size. Mesoporous silica was synthesized according to the literature[1] using a molecular ratio of the sol of 0.03 P123: 1.0 TEOS: 8.2 HCl: 300 H_2O. In 500 mL glass, 8.0 g of pluronic P123 (Poly(ethyleneglycol)) was dissolved in 250 mL 1.9 M $HCl_{aq.}$. The jar was sealed and heated to 40˚C with stirring for up to 3 hours. Then, 17 g of TEOS were added dropwise and the solution is stirred under this conditions for 24 hours. The mixture was transferred into an autoclave and reacted for another 24h at 100˚C. Then SBA-15 is filtered and washed, dried and calcined at 500˚C for 9h, applying a heat rate of 1˚C per minute.

Synthesis of MCM-41 with 3.0 nm pore size. The material was synthesized according to literature[2] using a molecular ratio of the sol of 0.11 CTAB: 1.0 TEOS: 0.11 NaOH: 111 H_2O. First, 4.05 g of cetyltrimethylammonium bromide (CTAB) was dissolved in 200 mL H_2O and stirred for 1 hour. Then, NaOH (440 mg) was added and the mixture was stirred for 15 more minutes. TEOS (20.6 mg) was added and the reaction mixture was stirred for 1 more hour. The reaction mixture was transferred in an autoclave and hydrothermally treated at 80˚C for 24 h. The next day, the mixture was filtered and washed with 0.1 M HCl in ethanol (3x 500 mL). The recovered material was dried at 130˚C for at least 24 hours and finally calcined at 500˚C for 9 hours applying a heat rate of 1˚C per hour.

Synthesis of MCM-41 with 2.5 nm pore size. The material was synthesized according to literature[3] using a molecular ratio of the sol of 0.14 CTAB: 1.0 TEOS: 1.7 NH_4NO_3: 150 H_2O. In a glass jar, cetyltetrammonium bromid (CTAB) (2.4 g) was dissolved in H_2O (120 mL). After 10 min, 30 wt% $NH_4OH_{(aq)}$ (10.5 mL) were added and stirred for another 15 min. Then, TEOS (10.5 mL) was slowly added and the mixture was stirred another hour. The precipitate was recovered by filtration without washing followed by hydrothermal treatment at 80 °C for 24 h. The material was dried in vacuo before using an extraction protocol[4] for removing the surfactant by NH_4^+/$CTMA^+$ exchange. Typically, 1 g of MCM-41 was dispersed in ethanol (95%) containing 0.3 g of NH_4NO_3 (150 mL). The mixture was stirred at 60˚C for 20 minutes. Then the solids were recovered by filtration and washed with cold ethanol, and the above treatment could be repeated twice. Finally, the MCM-41 was dried well. To be sure that the surfactant is

fully removed, the material was subsequently calcined at 550˚C for 3 hours with and heating rate of 1˚C per hour.

Dehydroxylation at 500˚C. All three materials SBA-15 (**1**) and MCM-41 (2 and **3**) with specific surface areas were dehydroxylated at 500°C for 24 h under dynamic vacuum (10^{-5} mbar) to generate SBA-15_{500} (**1**) and MCM-41_{500}, (**2**,**3**) respectively, as the well-ordered hexagonal structure is maintained under these conditions.[5]

Titration of Silanol. The concentration of silanol was determined by titration of Si-OH with MeLi.[6]

Passivation of external surface. One of our strategies was to perform external passivation.[7] Unfortunately, passivation is limited to a dehydroxylation temperature of only 200˚C as the trimethylsilyl groups decompose at 500˚C.[8, 9] A dehydroxylation temperature of 200˚C, however, would lead to a mixture of monopodal and bipodal SOMFs, which is not consistent with our study (MCM-41_{500}).

<u>Typical procedure of synthesis of WL_n@mesoporous silica$_{500}$</u>

Grafting of W(≡C*t*Bu)(CH_2*t*Bu)$_3$ and WMe_6 on SBA-15 and MCM-41, respectively were performed according to literature.[10, 11]

For **1A, 2A** and **3A**, **1, 2** or **3** (100-200 mg) were suspended in *n*-pentane (2 mL). Then, W(≡C*t*Bu)(CH_2*t*Bu)$_3$ (15 mg - 25 mg) dissolved in n-pentane (2 mL) were added. After complementation of the reaction (20 h), the supernatant was removed and the material was washed three times with fresh n-pentane. The sample was dried in high vacuo (10^{-5} mbar) and stored at -40˚C under Ar atmosphere in a glovebox.

For **1B**, **2B** and **3B**, **1, 2** or **3** (500 mg) were suspended in *n*-pentane (20 mL) in a double Schlenk. A solution of WMe_6 was added and reacted at -40˚C for 6h. Then, the solvent was removed by filtration, the precipitate was washed with *n*-pentane, until the remaining **B** was fully removed.

<u>Elemental analyses</u> were performed at Mikroanalytisches Labor Pascher (Germany). Additionally, W was determined on Varian 720-ES ICP-Optical Emission Spectrometer after the samples preparation by microwave digestions on and on Milestone ETHOS 1. Analysis of C and H was done on Flash 2000 Elemental Analyzer from Thermo Scientific.

The small-angle X-ray powder diffraction (XRD) data were acquired on a Bruker D8 advance diffractometer using Cu Kα monochromatic radiation (λ=1.054184Å) to confirm the hexagonal ordered structure of the samples.

Transmission-electron microscopy (TEM). The primary particle size and morphology of sample **3** was examined by conventional TEM. A microscope of model Titan, G 80-300 from FEI Company (Hillsboro, OR) was employed to carry out the analysis. Samples were loaded in argon-filled glove box onto a vacuum sealed holder of model 648 from Gatan, Inc. In this way, the samples were loaded into the microscope without their exposure to air. Conventional bright-field TEM (BF-TEM) as well as energy-filtered TEM (EFTEM) analyses were performed by operating the microscope at the accelerating voltage of 200 kV. X-ray energy dispersive (EDS) analysis for the determining the elemental composition had also been carried along with the BF-TEM analysis. The main objective of the EFTEM analysis was to generate the elemental maps of Si and W elements. This had been achieved by utilizing their Si L-edge (99 eV) and W-pre-O-edge (35 eV) present in the corresponding electron energy loss spectroscopy (EELS) datasets. It is to be noted that the 3-window method was utilized for generating the Si and W maps. The entire TEM analysis was carried out by using the Gatan Microscopy Suite of version GMS 2.3 from Gatan, Inc.
Structure of silica particles was found to be well preserved after their loading with W-containing ligand. Hence both channels and walls of the silica particles could be identified easily. The presence of W in the channels was proved by the W maps and Si-maps. It is imperative to note that the maximum intensity in the tungsten map cannot always be assigned to the regions having maximum tungsten in the channels (edge or center) for three reasons: 1) the applied energy loss of W at 35 eV is close to the zero-loss peak; 2) the pore size of the material is very small and 3) the sample might be slightly tilted. The white signal is presumed to emanating from the W ligand. The measurements on the white dot matched with channel width and hence it corroborates our argument that the W was present inside the channels.

Infrared spectra were recorded on a Nicolet 6700 FT-IR spectrometer equipped with a cell under controlled atmosphere.

Nitrogen adsorption–desorption isotherms at 77 K were measured using a Micromeritics ASAP 2024 physisorption analyzer. Specific surface areas were calculated following typical BET procedures. Pore size distribution was obtained using BJH pore analysis applied to the desorption branch of the nitrogen adsorption/desorption isotherm.

Solid State NMR (SS NMR). One-dimensional ^1H MAS and ^{13}C CP/MAS solid state NMR experiments were recorded on Bruker AVANCE III spectrometers operating at 400 MHz resonance frequencies for ^1H employing a conventional double-resonance 3.2 mm CP/MAS probe. In all cases the samples were packedinto zirconium rotors under an inert atmosphere inside gloveboxes. Dry nitrogen gas was utilized for sample spinning to prevent degradation of the samples. NMR chemical shifts are reported with respect to the external references TMS and adamantane. Potassium bromide (KBr) was used to calibrate the magic angle for the MAS probes. For ^{13}C CP/MAS NMR experiments, the following sequence was used: 90° pulse on the proton (pulse length 2.4 s), then a cross-polarization step with a contact time of typically 2 ms, and finally acquisition of the ^{13}C NMR signal under high-power proton decoupling. The delay between the scans was set to 5 s to allow the complete relaxation of the ^1H nuclei, and the number of scans of 60 000 for ^{13}C. An exponential apodization function corresponding to a line broadening of 80 Hz was applied prior to Fourier transformation.

A typical ^{13}C CP SS NMR spectra of **0A** is shown in Figure S1. Even after 60 000 scans (which corresponds to 84 h experimental time), the carbyne is not observed.[10]

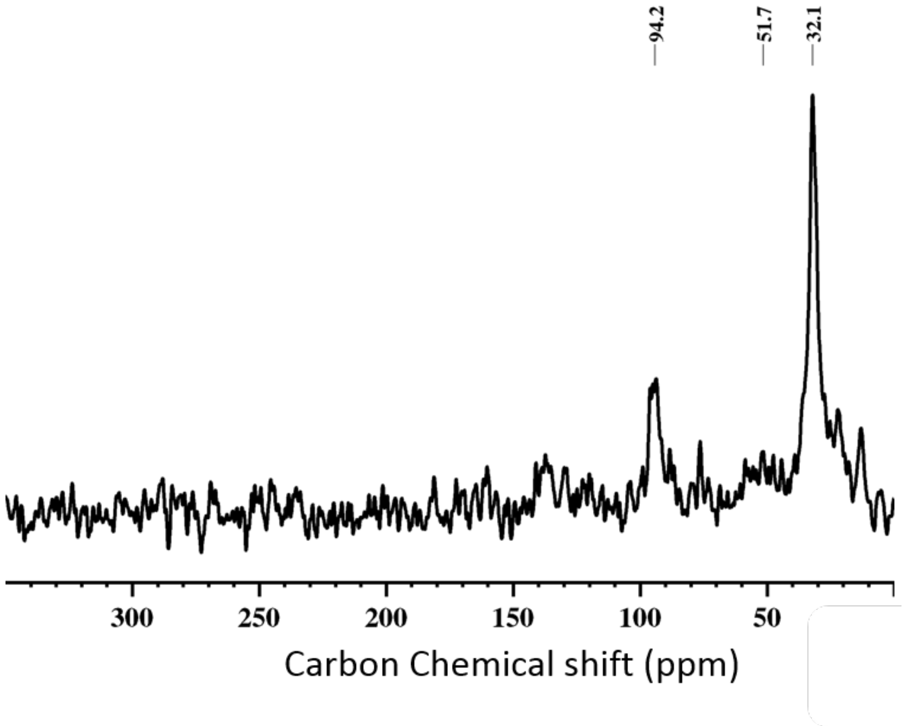

Figure S1. One dimensional (1D) ^{13}C CP/MAS NMR spectrum of ***0A*** *was recorded at room temperature at 100 MHz with 10 kHz MAS frequency and 60 000 scans.*

Dynamic Nuclear Polarization NMR spectroscopy (DNP SENS).

Sample Preparation. TEKPol was dried under high vacuum (10^{-4} mbar) and the solvents were stirred over calcium hydride and then distilled in vacuo. A radical solution consisting of 16 mM TEKPol (TEKPol, MW= 905 g/mol) in either 1,1,2,2-tetrachloroethane (TCE) or 1,2-dichlorobenzene (DCB) was used. The organometallic complexes $W(\equiv CtBu)(CH_2tBu)_3$ **A** and WMe_6 **B** are known to not react with chlorinated solvents.[11, 12] The SOMC catalysts were finely ground in a mortar and pestle prior to preparation of DNP experiments. DNP sample were prepared by incipient wetness impregnation. In a typical experiment, 15 mg (corresponding to the tungsten loading between 4-7 µmol) of the samples were impregnated with the appropriate volume of 16 mM solution of TEKPol (n_{TEKPol} = 0.5 - 1.2 µmol/sample) and packed into a 3.2 mm o.d. sapphire rotor capped with a teflon plug under argon atmosphere. The packed samples were then immediately or after a defined time inserted into the pre-cooled DNP probe for experiments.

Measurements: Data were acquired at Core Lab of King Abdullah University using 263 GHz/400 MHz Avance III Bruker DNP solid-state NMR spectrometer ($\nu_L(^{13}C)$ = 100.6 MHz) equipped with a 3.2 mm Bruker triple resonance low temperature magic angle spinning (LTMAS) probe and the experiments were performed at ca. 100 K with a 263 GHz gyrotron. The sweep coil of the main magnetic field was set for the microwave irradiation occurring at the ^1H positive enhancement maximum of the TEKPol biradical.

^{13}C DNP SENS. For ^{13}C NMR experiments the acquisition parameters used are 3 s repetition delay, a ^1H π/2 pulse length of 2.5 µs to afford 100 kHz ^1H decoupling using the SPINAL 64 method. The contact time was typically 2 ms for cross polarization experiments. The MAS frequency varied between 8 and 10 kHz. The two-dimensional (2D) ^1H-^{13}C heteronuclear correlation (HETCOR) spectra was acquired with 208 scans per t_1 increment, 96 individual increments and 0.2 ms contact time. During t_1, e-DUMBO-1 homonuclear ^1H decoupling was applied and proton chemical shift were corrected by applying a scaling factor of 0.57.[13] All ^{13}C NMR spectra were referenced to adamantane with the higher frequency peak set to 38.48 ppm with respect to TMS (0 ppm). More detailed information can be obtained from Table S1.

Processing: DNP enhancements were determined by comparing the integration of the resonance of interest for the spectra acquired with and without microwave. Proton and carbon spin lattice relaxation measurements were measured using saturation recovery experiment. Data are fit using either a stretched-exponential of the form: $S(\tau) = A\left[1 - \exp(-(\frac{\tau}{T_1^*})^{\beta})\right]$, or a mono-exponential of the form: $(\tau) = A\left[1 - m\exp(-\frac{\tau}{T_1^*})\right]$; where, A is the equilibrium signal intensity with microwave irradiation, S(τ) is

the integrated intensity at recycle delay time τ, β is the stretching parameter and T_1^* is the build-up value.

Table S1. Acquisition parameters for the double resonance probe.

Pulse Sequence	Hahn-Echo	CP MAS	HETCOR	CP MAS
**Nuclei**	^{1}H	^{13}C	^{1}H-^{13}C	^{29}Si
**µwave on/ µwave off**	µwave on/ µwave off			
**Number of Scans**	16	512-8192	208	1024/10240
**Recycle Delay (s)**	3	3	3	3
**Dwell (µs)**	6.5	16.8	16.8	12.0
**Spectral width (kHz)**	10	10	10	10
**Spinning Speed (kHz)**	8.0	8.0-12.0	8.0	8.0
**Acquisition length (number of points)**	1024	2048	2048	2018
**^{1}H 90 degree pulse width (π/2) (µs)**	2.5	2.3	2.3	2.3
**Contact pulse length (ms)**	-	3	0.2	5
**^{1}H rf field during contact pulse (kHz)**	-	100	100	100
**X rf field during contact pulse (kHz)**	100	100	100	100
**^{1}H rf field during SPINAL-64 decoupling (kHz)**	-	100	100	100

2. Nitrogen adsorption/desorption isotherms

Nitrogen adsorption/desorption isotherms of **1**, **2** and **3** are shown in Figure S2 and Figure S3. The isotherms can be classified as type IV isotherms with a narrow pore size distribution. Furthermore, structural parameters of the mesoporous material were determined and are summarized in Table S2.

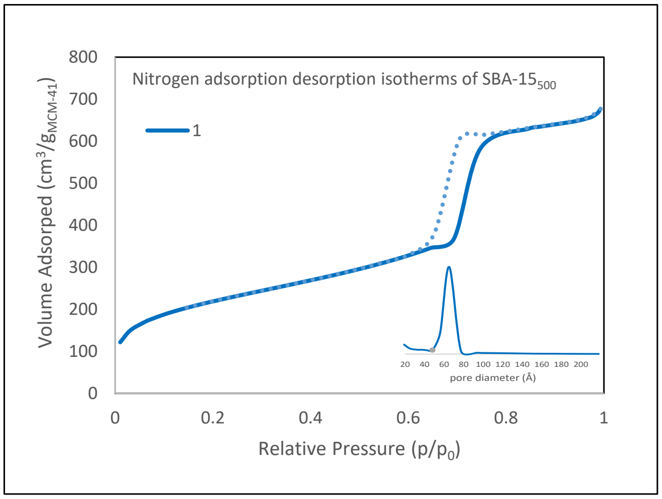

Figure S2. Nitrogen adsorption/desorption isotherm at 77 K of 1 including the pore size distribution according to the BJH Method.

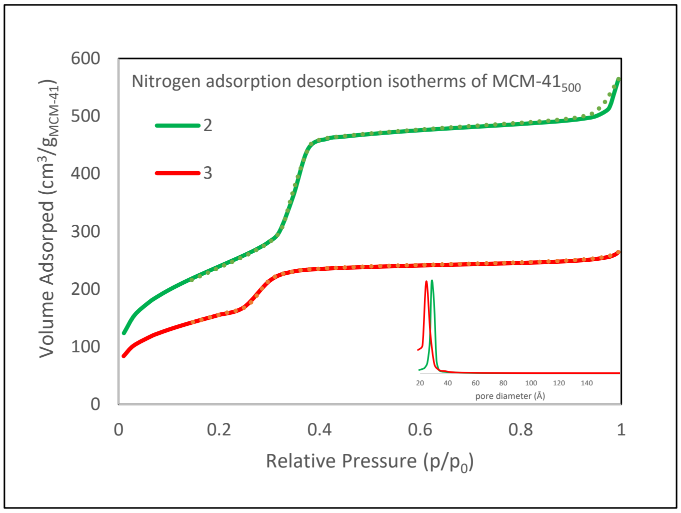

Figure S3. Nitrogen adsorption/desorption isotherm at 77 K of 2 and 3 including the pore size distribution according to the BJH method.

*Table S2. Textural parameters of **1, 2** and **3** from Nitrogen sorption combined with small angle X-ray diffraction*

mesoporous silica	S_{BET} ($m^2 \cdot g^{-1}$)	$S_{BET, ext}$ [a] ($m^2 \cdot g^{-1}$)	V_P [b] ($cm^3 \cdot g^{-1}$)	D_P [c] (Å)	d_{100} [d] (Å)	a_0 [e] (Å)	Wall thickness[f] (Å)
**$SBA\text{-}15_{500}$ 6.0 nm (1)**	758	68	1.098	60.1	98.1	113.2	53.2
**$MCM\text{-}41_{500}$ 3.0 nm (2)**	882	76	0.865	29.7	44.7	51.6	21.9
**$MCM\text{-}41_{500}$ 2.5 nm (3)**	555	35	0.360	25.1	37.8	43.6	18.5

[a]$S_{BET, ext}$ represents the external surface area, calculated from the plateau of the isotherm; [b]Total pore volume at p/p_0 = 0.96; [c]Pore size from desorption branch applying the BJH pore analysis between p/p_0 =0.05 and 0.9; [d]*d*(100) spacing; [e]a_0= 2d(100)/√3, hexagonal lattice parameter calculated from XRD; [f]Calculated by a0 – D_p.

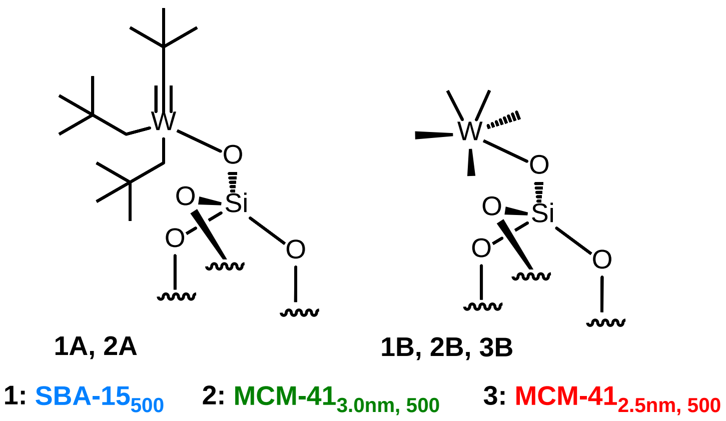

Figure S4. Nomenclature for SBA15 and MCM41 samples with 3.0 nm and 2.5 nm

*Table S3. Analytical data for **1A-3B***

mesoporous silica	[≡SiOH] (mmol/g)	W wt.%	C wt.%	H wt.%	C/W (th.)	H/C (th.)
**1A**	2.9	6.1	5.9	1.1	14.8 (15)	2.2 (2.1)
**1B**	2.9	5.7	2.4	0.62	6.3 (5)	3.2 (3.0)
**2A**	2.4	4.8	4.6	0.87	14.7 (15)	2.3 (2.1)
**2B**	2.4	6.2	2.2	0.54	5.4 (5)	3.0 (3.0)
**3B**	3.3	4.0	1.5	0.47	5.7 (5)	3.7 (3.0)

3. IR spectra

The IR spectra of these materials obtained after reaction are quite similar. Each one representative of **A** and **B** can be found in Figure S5 and Figure S6. For **A** (by the example of **1A**), a strong decrease in the intensity of OH vibrational band at 3741 cm^{-1} is observed. New vibrational and stretching bands in the region between 3023-2788 cm^{-1} of neopentyl moieties appear at 2958 [$\upsilon_{as}(CH_3)$], 2866 [$\nu_s(CH_2)$], 1469 [$\delta_{as}(CH_3)$], and 1361 cm^{-1} [δ_s (CH_3)] as it was already described in literature.

Similarly, for **B** (by the example of **2B**), the intensity of the OH vibrational and stretching bands at 3741 cm^{-1} is observed. New vibrational bands of the W-methyl ligand moieties appear in the region between 3077-2784 cm^{-1}, with bands at 3016, 2985, 2946 and 2881 cm^{-1}.

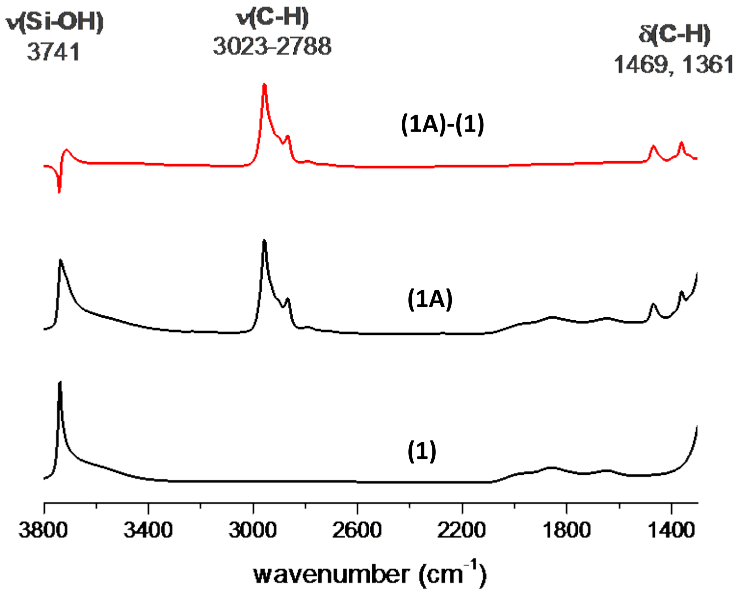

*Figure S5. Representative FT-IR spectrum **1, 1A** and subtraction **1A-1** in red.*

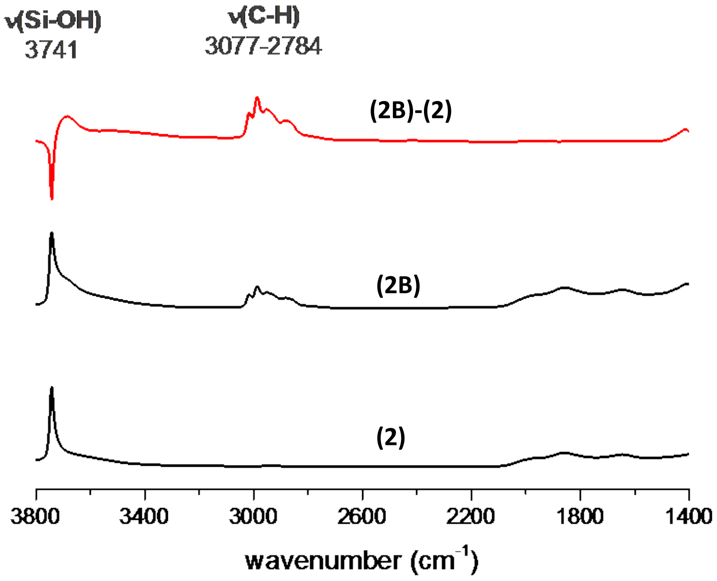

*Figure S6. Representative FT-IR spectrum **2**, **2B** and subtraction **2B**-**2** in red.*

4. DNP SENS

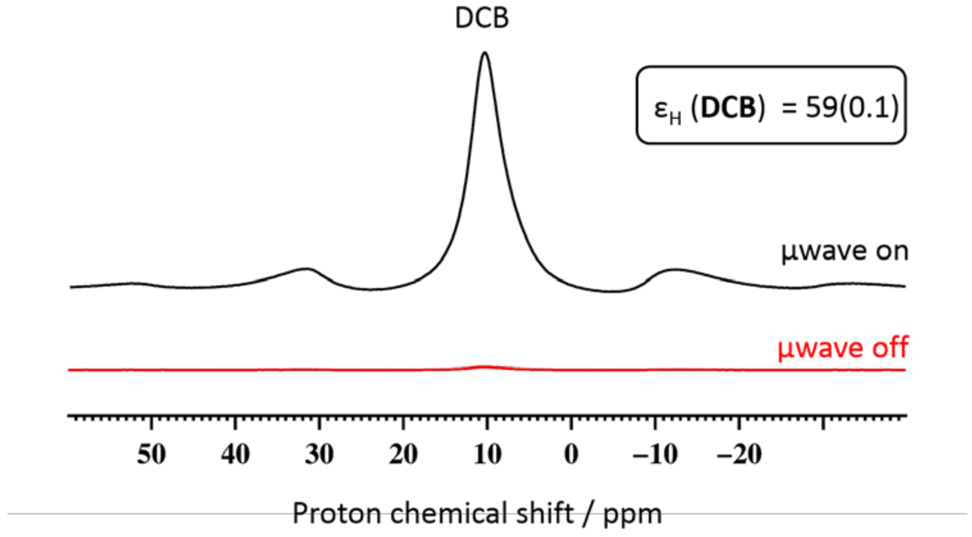

Figure S7. DNP SENS ^{1}H MAS spectrum (100 K, 400 MHz / 263 GHz) of **2B** *in 16 mM TEKPol solution in DCB. The recycle delay was 3 s and the MAS frequency was 8 kHz. The red line represents the spectrum without microwave irradiation (16 scans), the black line shows experiment with microwave irradiation (16 scans).*

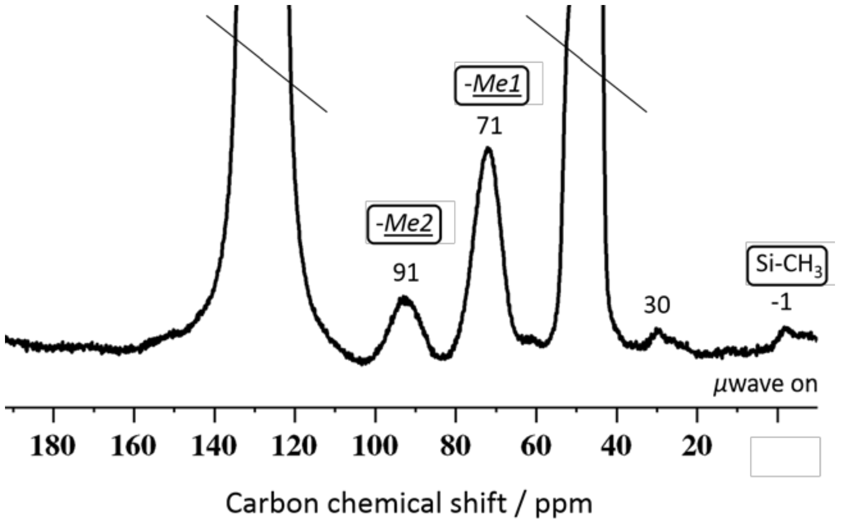

Figure S8. DNP SENS ^{13}C CP-MAS spectrum (100 K, 400 MHz / 263 GHz) of ***2B*** *in 16 mM TEKPol solution in DCB in the range between 190 to -10 ppm.. The spectrum was recorded with 512 scans. The recycle delay was 3 s, the contact time was 3 ms and the MAS frequency was 8 kHz. The signal at 30 ppm corresponds to reacted TEKPol.*

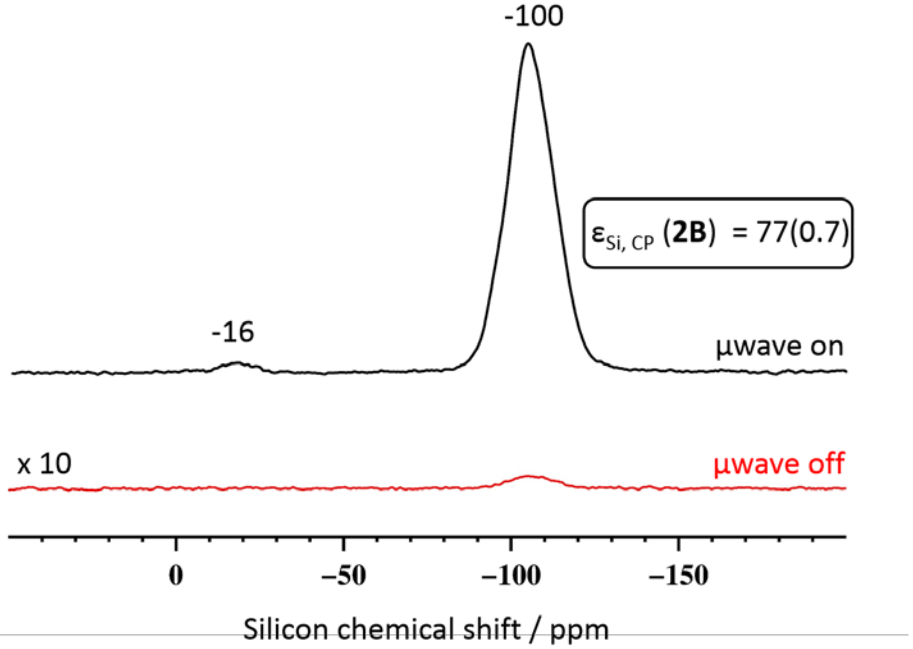

Figure S9. DNP SENS ^{29}Si CP-MAS spectrum (100 K, 400 MHz / 263 GHz) of ***2B*** *in 16 mM TEKPol solution in DCB. The recycle delay was 3 s, the contact time was 5 ms and the MAS frequency was 8 kHz. The red line represents the spectrum without microwave irradiation (10240 scans), the black line shows the spectrum with microwave irradiation (1024 scans).*

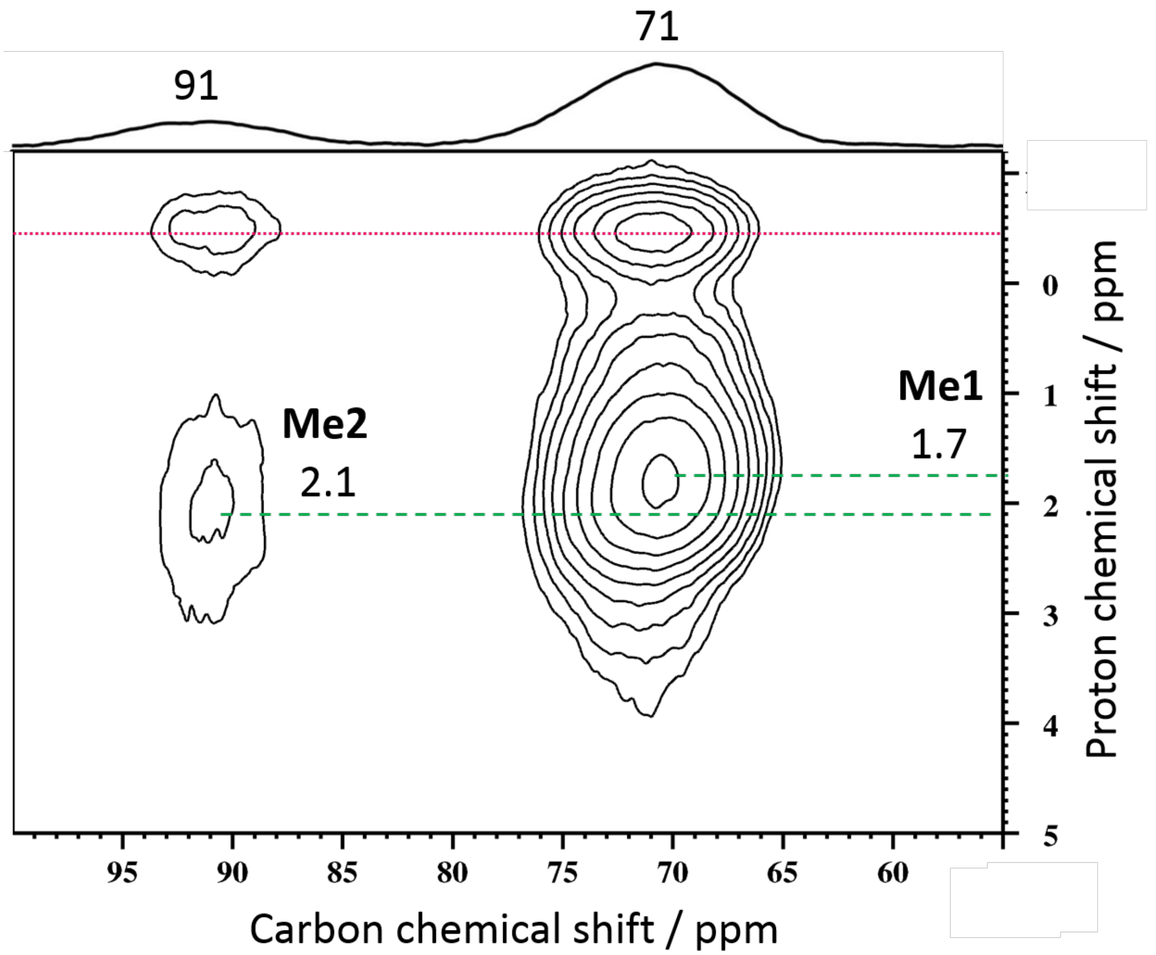

*Figure S10. ^{1}H-^{13}C HETCOR DNP SENS (100 K, 400 MHz / 263 GHz) of **2B** in 16 mM TEKPol solution in DCB. (The red line indicates the transmitter spike)*

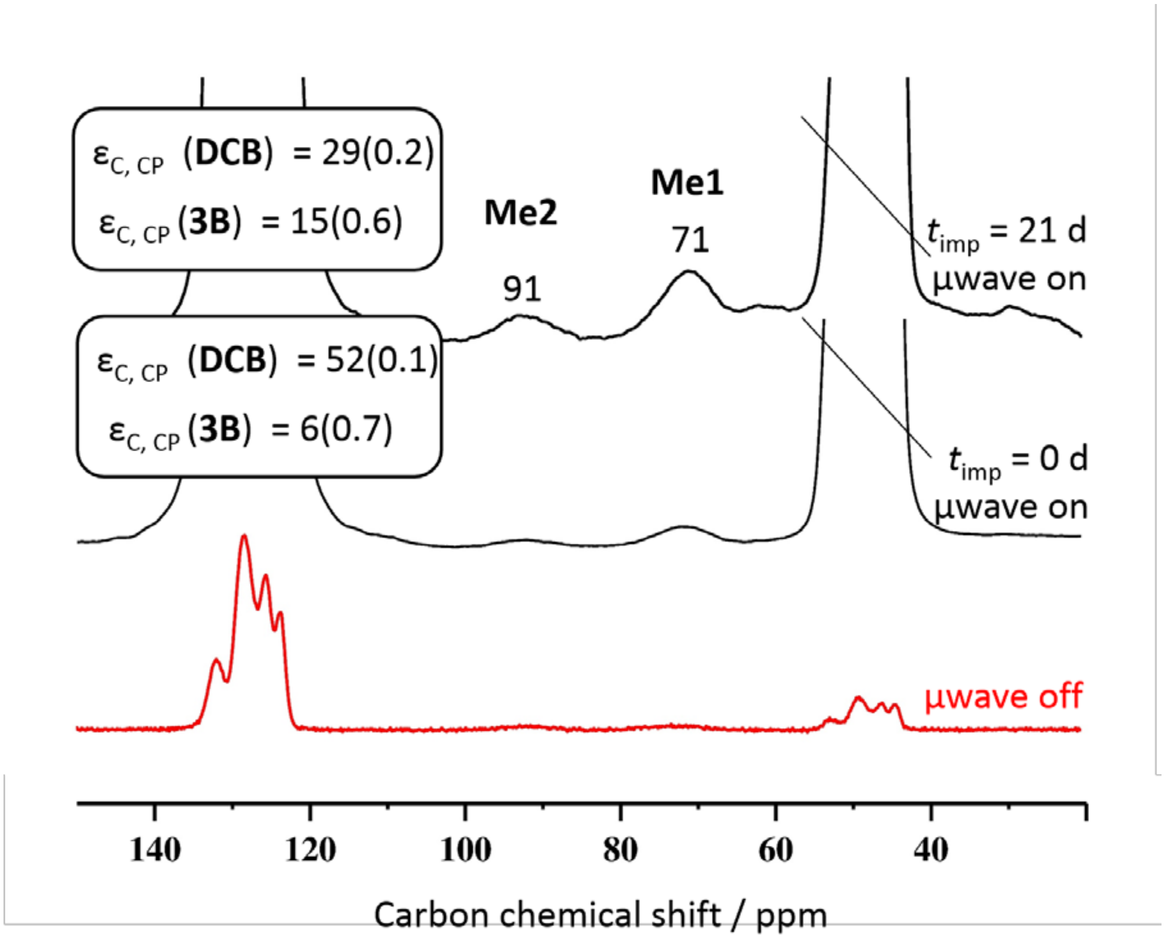

*Figure S11. ^{13}C CPMAS DNP SENS (100 K, 400 MHz / 263 GHz) of **3B** in 16 mM TEKPol solution in DCB. The red trace corresponds to the spectrum (1088 scans) without microwave, the black trace (1088 scans) with microwave irradiation recorded after t_{imp} = 0 d and the black trace to the spectrum (2048 scans) with microwave irradiation recorded after storing the rotor for 21 days at -4˚C.*

Table S4. ^{1}H, ^{13}C and ^{29}Si enhancements and respective time savings ($T_{ON/OFF}$) of the surface complexes **0A**, **1A**, **1B**, **2A**, **2B** *and* **3B** *impregnated with a 16 mM TEKPol solution in either DCB or TCE. The spectra were recorded with a recycle delay of 3 s after sample preparation if not noted differently.*

sample	Solvent	ε_H (solvent)	$\varepsilon_{C\ CP}$ (solvent)	$\varepsilon_{C\ CP}$ (surface)	$T_{ON/OFF}$ [a] (surface)	$\varepsilon_{Si\ CP}$ (surface)	$T_{ON/OFF}$ [b] (surface)
**0A**	DCB	5(0.4)	4(0.3)	-	-	-	-
**1A**[b]	DCB	2(0.03)	2(0.1)	-	-	-	-
**1B**	DCB	13(0.07)	7(0.3)	-	-	-	-

**2A**	DCB	11(1.5)	12(0.5)	-	-	-	-
**2A**	TCE	29(1.1)	5(0.2)	-	-	-	-
**2B**	DCB	59(0.2)	42(0.3)	31(0.2)	961(0.1)	77(0.7)	5929(0.5)
**3B**	DCB	85(0.8)	52(0.2)	6(0.7)	36(0.5)	-	-
**3B**[d]	DCB	20(0.6)	29(0.2)	15(0.6)	225(0.4)	-	-

[a]Time savings to obtain a ^{13}C CP SS NMR spectrum with the same quality as obtained by this DNP SENS experiment; [b]Time savings to obtain a ^{29}Si CP SS NMR spectrum with the same quality as obtained by this DNP SENS experiment; [c]The sample was left to impregnate at -4˚C for 4 hours; [d]The sample was left to impregnate at -4˚C for 21 days.

*Table S5. Summary of ^{13}C relaxation measurements for **2B** with 16 mM TEKPol in DCB acquired with microwave irradiation.* [a]

	**Me1**	**Me2**
$\delta(^{13}C)$ / ppm	71(24)	91(4)
A **(au)**	0.938(0.067)	0.919(0.031)
T_1^* **(s)**	27(11)	9(2)
β	0.466(0.078)	0.647(0.098)

[a] Data are fit using a stretched-exponential, where, *A* is the equilibrium signal intensity with microwave irradiation, *S*(τ) is the integrated intensity at polarization time of τ, *β* is the stretching parameter. The full width at half height for the two chemical shifts are given in parentheses for δ.

Table S6. Summary of ^1H relaxation measurements of **3B** with 16 mM TEKPol in DCB *acquired with microwave irradiation*. [a]

	**0 d**	**1 d**	**2 d**	**3 d**	**4 d**	**5 d**	**6 d**
**A (au)**	1.25(0.04)	1.05(0.04)	0.97(0.04)	0.97(0.04)	0.98(0.03)	0.98(0.04)	0.98(0.04)
T_1^***(s)**	98(10)	222(20)	313(33)	319(33)	307(33)	306(36)	300(37)
m	0.964(0.001)	0.967(0.001)	0.961(0.001)	0.964(0.001)	0.951(0.001)	0.944(0.001)	0.945(0.001)

[a] Parameters where fit with a mono-exponential and error bounds are in parenthesis.

*Table S7. Summary of ¹H and ¹³C enhancements of compound **3B*** at each time point of the impregnation measurements.[a]

days	ε_H solvent	$\varepsilon_{C, CP}$ solvent
**0**	84(1.4)	37(0.09)
**1**	49(4.3)	55(0.30)
**2**	26(0.4)	31(0.03)
**3**	45(1.3)	28(0.02)
**4**	63(0.2)	49(0.01)
**5**	37(0.4)	15(0.01)
**6**	37(0.1)	46(0.04)

[a]Error bounds are in parenthesis. Slight differences in glass formation may contribute to variability of enhancement when the sample is frozen at different times.

5. DFT calculations

To estimate the size of TEKPol, W(≡C*t*Bu)(CH_2*t*Bu)$_3$ **A** and WMe_6 **B** (see Figure S12), calculations were performed with the generalized gradient approximation (GGA) functional with the Gaussian09 software using the BP86 level of theory of Becke and Perdew.[14-16] The electronic configuration of the molecular systems are described with the standard split-valence basis set with a polarization function of Ahlrichs and co-workers for H, C, N and O (SVP keyword in Gaussian).[17] For W, the small-core, quasi-relativistic Studgard/Dresden effective core potential, with an associated contracted valence basis set (standard SDD keyword in Gaussian09) was used.[18, 19]

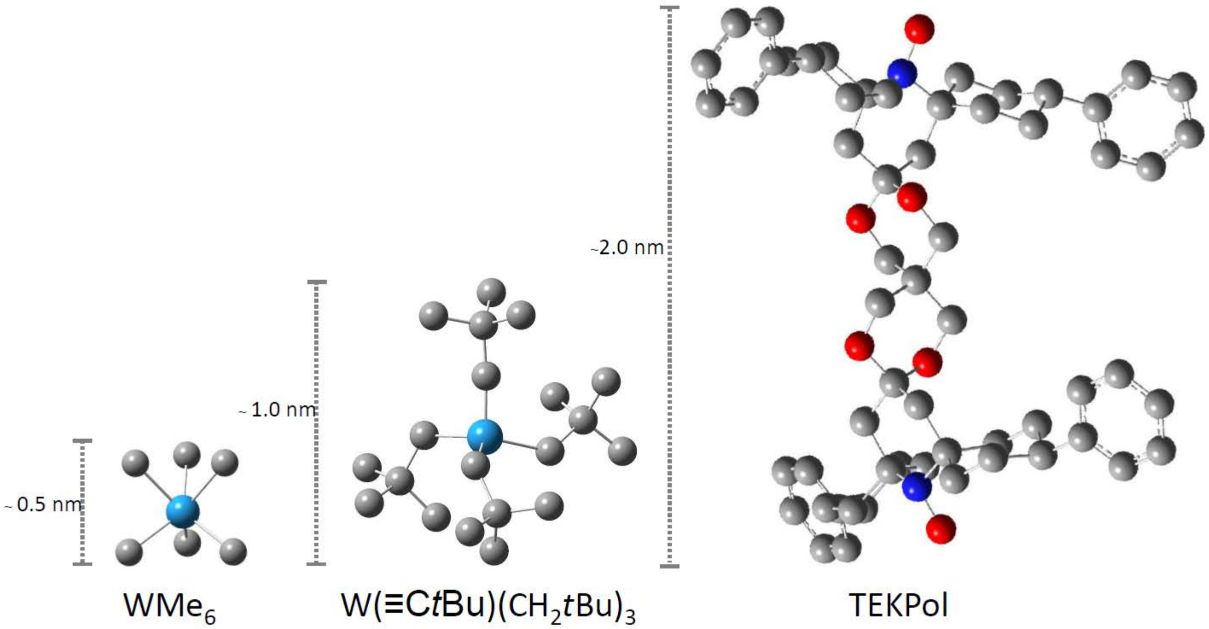

*Figure S12. DFT optimized structures of TEKPol, **A** and **B** including their length in nm.*

The results for TEKPol are in accordance with results from literature[20] having an oxygen-oxygen-distance $d_{O\text{-}O}$ = 13.46 Å ($d_{O\text{-}O(DFT)}$ = 13.84 Å) and a nitrogen-nitrogen distance $d_{N\text{-}N}$ = 11.09 Å ($d_{N\text{-}N(DFT)}$ = 11.47 Å). The coordinates for **B**, were taken from literature.[11] The coordinates can be found in Table S8, Table S9 and Table S10.

Table S8. XYZ coordinates of TEKPol from the DFT optimization

TEKPol SCF Done: -2850.30440923 A.U.			
C	1.251621	0.507027	-0.181424
C	0.252383	-1.255593	1.242941
H	1.282064	1.284732	0.622880
H	1.227127	1.044578	-1.154267
H	-0.526400	-2.040206	1.319112
H	0.191961	-0.625191	2.165292
O	2.445953	-0.269132	-0.195491

O	1.500837	-1.934866	1.156223
C	-0.000013	-0.371018	0.000088
C	-0.252452	-1.255213	-1.243021
H	-0.192018	-0.624540	-2.165186
H	0.526300	-2.039834	-1.319431
C	-1.251599	0.507043	0.181858
H	-1.281993	1.284994	-0.622212
H	-1.227079	1.044292	1.154869
O	-2.445970	-0.269049	0.195686
O	-1.500939	-1.934454	-1.156494
C	-2.649082	-1.081420	-0.970951
C	-2.946212	-0.271171	-2.249617
C	-3.810798	-2.035595	-0.695425
H	-2.887841	-1.008308	-3.078698
H	-2.153670	0.481505	-2.444998
H	-3.752769	-2.800749	-1.497444
H	-3.603740	-2.553511	0.264664
C	2.946251	-0.271961	2.249798
C	3.810678	-2.036024	0.695132
C	2.649036	-1.081836	0.970910
H	2.887787	-1.009294	3.078699
H	2.153813	0.480773	2.445372
H	3.603589	-2.553651	-0.265105
H	3.752560	-2.801398	1.496934
N	-5.405856	-0.551881	-1.909117
N	5.405859	-0.552878	1.909325
O	6.645247	0.027035	1.939100
O	-6.645244	0.028040	-1.938822
C	-4.331791	0.414094	-2.302391
C	-4.679628	0.763632	-3.781221
C	-4.416392	1.726384	-1.466045
C	-3.945777	1.972155	-4.373558

H	-5.778551	0.937250	-3.751980
H	-4.519376	-0.147056	-4.400991
C	-3.715031	2.952588	-2.072091
H	-5.517085	1.896864	-1.388579
H	-4.033315	1.553255	-0.440351
C	-4.173430	3.243353	-3.520077
H	-4.289435	2.153755	-5.417257
H	-2.848418	1.780224	-4.446577
H	-3.893822	3.849913	-1.435406
H	-2.606697	2.812380	-2.079212
H	-5.273886	3.417439	-3.485165
C	-5.223895	-1.415710	-0.699555
C	-5.570310	-0.649443	0.611977
C	-6.283091	-2.541327	-0.893093
C	-5.866900	-1.533255	1.834794
H	-6.465868	-0.048904	0.325104
H	-4.752608	0.052451	0.867108
C	-6.541265	-3.430348	0.328665
H	-7.201543	-1.985885	-1.186609
H	-5.986143	-3.150370	-1.776434
C	-6.962214	-2.589652	1.558373
H	-6.171086	-0.898857	2.699685
H	-4.941665	-2.062091	2.166413
H	-7.333416	-4.178390	0.096987
H	-5.632964	-4.023038	0.591817
H	-7.886838	-2.036817	1.271388
C	4.331909	0.413134	2.302725
C	4.679730	0.762479	3.781597
C	4.416718	1.725492	1.466513
C	3.945990	1.971045	4.373993
H	5.778673	0.936004	3.752415
H	4.519343	-0.148237	4.401288

C	3.715454	2.951725	2.072609
H	5.517439	1.895855	1.389163
H	4.033715	1.552453	0.440773
C	4.173831	3.242305	3.520639
H	4.289615	2.152506	5.417727
H	2.848600	1.779254	4.446925
H	3.894375	3.849072	1.435990
H	2.607106	2.811619	2.079652
H	5.274307	3.416263	3.485786
C	5.223847	-1.416288	0.699488
C	5.570346	-0.649561	-0.611755
C	6.282915	-2.542105	0.892582
C	5.866731	-1.532929	-1.834938
H	6.466016	-0.049287	-0.324677
H	4.752756	0.052584	-0.866568
C	6.540932	-3.430675	-0.329539
H	7.201437	-1.986896	1.186314
H	5.985911	-3.151463	1.775687
C	6.961898	-2.589589	-1.559000
H	6.170959	-0.898224	-2.699592
H	4.941383	-2.061468	-2.166705
H	7.333009	-4.178903	-0.098218
H	5.632552	-4.023153	-0.592892
H	7.886643	-2.037012	-1.271912
C	-3.535628	4.490455	-4.108114
C	-4.324055	5.603355	-4.480877
C	-2.131370	4.587877	-4.309264
C	-3.753058	6.765998	-5.027007
H	-5.416875	5.549119	-4.333998
C	-1.554227	5.747206	-4.853148
H	-1.482153	3.741753	-4.031344
C	-2.356130	6.845197	-5.216787

H	-4.399321	7.615866	-5.304442
H	-0.460995	5.794806	-4.993592
H	-1.899129	7.753910	-5.642075
C	-7.298099	-3.431257	2.777988
C	-6.334786	-4.284259	3.384852
C	-8.589185	-3.397449	3.352922
C	-6.658039	-5.057864	4.511899
H	-5.316568	-4.337352	2.966369
C	-8.919527	-4.169778	4.480376
H	-9.352575	-2.742856	2.897323
C	-7.949119	-5.009576	5.070311
H	-5.888966	-5.708335	4.962562
H	-9.937188	-4.116397	4.902838
H	-8.198119	-5.617880	5.955612
C	7.297557	-3.430916	-2.778871
C	6.333937	-4.283290	-3.386129
C	8.588723	-3.397496	-3.353654
C	6.656993	-5.056725	-4.513345
H	5.315630	-4.336021	-2.967817
C	8.918865	-4.169648	-4.481286
H	9.352342	-2.743354	-2.897789
C	7.948170	-5.008861	-5.071577
H	5.887685	-5.706715	-4.964300
H	9.936599	-4.116578	-4.903613
H	8.197012	-5.617032	-5.957012
C	3.536171	4.489417	4.108804
C	4.324731	5.602248	4.481544
C	2.131952	4.586949	4.310123
C	3.753881	6.764889	5.027808
H	5.417525	5.547941	4.334516
C	1.554954	5.746280	4.854148
H	1.482629	3.740907	4.032194

C	2.356982	6.844191	5.217769
H	4.400242	7.614695	5.305217
H	0.461741	5.793953	4.994716
H	1.900101	7.752899	5.643194

*Table S9. XYZ coordinates of W(≡CtBu)(CH_2tBu)$_3$ **A** after DFT optimization*

W(≡C*t*Bu)(CH_2*t*Bu)$_3$ **A** SCF Done: -853.997327558 A.U.			
C	-0.721475	0.062174	1.862853
H	-0.561660	-1.023282	2.121758
H	0.080829	0.615487	2.425719
C	0.754450	1.867752	-0.898058
H	0.633029	1.925471	-2.003079
H	-0.097013	2.482016	-0.492785
C	1.250965	-1.761594	-0.609801
H	0.712644	-2.499545	-1.241184
H	1.920982	-1.201364	-1.327817
C	-1.286954	-0.261815	-1.188229
C	-2.364826	-0.492364	-2.206673
C	-3.466991	0.590872	-2.082224
H	-4.248044	0.441740	-2.859187
H	-3.960039	0.550206	-1.089697
H	-3.041375	1.607272	-2.211744
C	-1.727739	-0.416067	-3.620723
H	-0.927415	-1.175415	-3.737924
H	-2.496042	-0.598071	-4.403509
H	-1.280895	0.582938	-3.802590
C	-2.983768	-1.899315	-1.997623
H	-3.444307	-1.988661	-0.992650
H	-3.771176	-2.094300	-2.757832
H	-2.211680	-2.690593	-2.088956
C	2.129786	-2.529284	0.423405

C	1.237035	-3.486570	1.244845
H	1.837739	-4.065769	1.978510
H	0.457302	-2.933951	1.810855
H	0.717858	-4.212783	0.584354
C	2.861666	-1.561960	1.380961
H	3.511668	-2.114347	2.093252
H	3.512031	-0.853729	0.824160
H	2.149613	-0.967156	1.995849
C	3.186972	-3.368303	-0.336337
H	3.813337	-3.960768	0.366068
H	2.701343	-4.078123	-1.038770
H	3.865915	-2.718242	-0.928468
C	2.105319	2.528947	-0.491409
C	2.080832	4.015428	-0.926991
H	3.035379	4.527741	-0.675210
H	1.926255	4.107486	-2.023044
H	1.257819	4.566992	-0.424413
C	3.279181	1.826844	-1.209648
H	4.248591	2.314132	-0.969605
H	3.361981	0.758787	-0.915948
H	3.149398	1.859671	-2.312519
C	2.318881	2.466445	1.037225
H	2.396486	1.418382	1.400759
H	3.257848	2.979977	1.336407
H	1.482205	2.952531	1.583322
C	-2.105628	0.515569	2.397246
C	-2.071423	0.537742	3.945744
H	-1.825250	-0.464683	4.356989
H	-1.309026	1.253126	4.322769
H	-3.054555	0.841894	4.366184
C	-3.203081	-0.463278	1.934349
H	-4.206760	-0.128096	2.272757

H	-3.213483	-0.543342	0.828772
H	-3.031410	-1.481713	2.345052
C	-2.421222	1.936978	1.879323
H	-2.490691	1.944541	0.771475
H	-3.385112	2.309429	2.288485
H	-1.631090	2.659423	2.179012
W	0.095201	-0.021007	-0.097095

*Table S10. XYZ coordinates of WMe_6 **B** after DFT optimization*

WMe_6 **B**SCF Done: -306.417017440 A.U.			
W	0.089954	0.000541	-0.000878
C	-1.478339	1.525938	-0.331081
H	-1.312668	2.328682	0.423010
H	-1.318753	1.970070	-1.339247
H	-2.528579	1.186887	-0.257646
C	-1.456391	-0.478191	1.507647
H	-2.511050	-0.371370	1.192478
H	-1.287364	-1.532748	1.823971
H	-1.284351	0.172705	2.394288
C	-1.474915	-1.061546	-1.149207
H	-1.321213	-0.801573	-2.221132
H	-1.300439	-2.155291	-1.037509
H	-2.525609	-0.843172	-0.882134
C	1.233922	-0.520038	1.742224
H	1.079773	-1.556157	2.110102
H	2.293543	-0.433093	1.400305
H	1.095793	0.177571	2.595502
C	1.209878	1.784541	-0.427701
H	2.275130	1.459500	-0.346879
H	1.053482	2.177818	-1.454692
H	1.052369	2.614755	0.292246

C	1.213923	-1.254916	-1.335095
H	1.036140	-1.071356	-2.415303
H	2.278252	-0.998084	-1.115009
H	1.080480	-2.339923	-1.138125

1 D. Y. Zhao, Q. S. Huo, J. L. Feng, B. F. Chmelka and G. D. Stucky, *Journal of the American Chemical Society,* 2014, **136**, 10546.

2 S. W. Choi and H. K. Bae, *Ksce Journal of Civil Engineering,* 2014, **18**, 1977.

3 A. Berenguer-Murcia, J. Garcia-Martinez, D. Cazorla-Amoros, A. Martinez-Alonso, J. M. D. Tascon and A. Linares-Solano, F. RodriguezReinoso, B. McEnaney, J. Rouquerol,K. Unger, 2002, 144, 83.

4 N. Lang and A. Tuel, *Chemistry of Materials,* 2004, **16**, 1961.

5 G. Cu, P. P. Ong and C. Chu, *Journal of Physics and Chemistry of Solids,* 1999, **60**, 943.

6 S. C. Antakli and J. Serpinet, *Chromatographia,* 1987, **23**, 767.

7 J. D. Webb, T. Seki, J. F. Goldston, M. Pruski and C. M. Crudden, *Microporous and Mesoporous Materials,* 2015, **203**, 123.

8 S. D. Bhagat, Y.-H. Kim, K.-H. Suh, Y.-S. Ahn, J.-G. Yeo and J.-H. Han, *Microporous and Mesoporous Materials,* 2008, **112**, 504.

9 M. L. Pena, V. Dellarocca, F. Rey, A. Corma, S. Coluccia and L. Marchese, *Microporous and Mesoporous Materials,* 2001, **44**, 345.

10 E. Le Roux, M. Taoufik, M. Chabanas, D. Alcor, A. Baudouin, C. Coperet, J. Thivolle-Cazat, J. M. Basset, A. Lesage, S. Hediger and L. Emsley, *Organometallics,* 2005, **24**, 4274.

11 M. K. Samantaray, E. Callens, E. Abou-Hamad, A. J. Rossini, C. M. Widdifield, R. Dey, L. Emsley and J. M. Basset, *Journal of the American Chemical Society,* 2014, **136**, 1054.

12 R. R. Schrock, D. N. Clark, J. Sancho, J. H. Wengrovius, S. M. Rocklage and S. F. Pedersen, *Organometallics,* 1982, **1**, 1645.

13 B. Elena, G. de Paëpe and L. Emsley, *Chemical Physics Letters,* 2004, **398**, 532.

14 A. D. Becke, *Physical Review A,* 1988, **38**, 3098.

15 J. P. Perdew, *Phys. Rev. B,* 1986, **33**, 8822.

16 J. P. Perdew, *Phys. Rev. B,* 1986, **34**, 7406.

17 A. Schafer, H. Horn and R. Ahlrichs, *Journal of Chemical Physics,* 1992, **97**, 2571.

18 U. Haussermann, M. Dolg, H. Stoll, H. Preuss, P. Schwerdtfeger and R. M. Pitzer, *Molecular Physics,* 1993, **78**, 1211.

19 T. Leininger, A. Nicklass, H. Stoll, M. Dolg and P. Schwerdtfeger, *The Journal of Chemical Physics,* 1996, **105**, 1052.

20 A. Zagdoun, G. Casano, O. Ouari, M. Schwaerzwalder, A. J. Rossini, F. Aussenac, M. Yulikov, G. Jeschke, C. Coperet, A. Lesage, P. Tordo and L. Emsley, *Journal of the American Chemical Society,* 2013, **135**, 12790.
